# D-dimer and high-sensitivity C-reactive protein levels to predict venous thromboembolism recurrence after discontinuation of anticoagulation for cancer-associated thrombosis

**DOI:** 10.1038/s41416-018-0269-5

**Published:** 2018-10-15

**Authors:** Luis Jara-Palomares, Aurora Solier-Lopez, Teresa Elias-Hernandez, Maria Isabel Asensio-Cruz, Isabel Blasco-Esquivias, Veronica Sanchez-Lopez, Maria Rodriguez de la Borbolla, Elena Arellano-Orden, Lionel Suarez-Valdivia, Samira Marin-Romero, Lucia Marin-Barrera, Aranzazu Ruiz-Garcia, Emilio Montero-Romero, Silvia Navarro-Herrero, Jose Luis Lopez-Campos, Maria Pilar Serrano-Gotarredona, Juan Manuel Praena-Fernandez, Jose Maria Sanchez-Diaz, Remedios Otero-Candelera

**Affiliations:** 1 Medical Surgical Unit of Respiratory Diseases, Virgen del Rocio Hospital, Seville, Spain; 2 Centro de Investigación Biomédica en Red de Enfermedades Respiratorias (CIBERES), Instituto de Salud Carlos III, Madrid, Spain; 3 Internal Medicine, Emergency Service, Virgen Macarena Hospital, Seville, Spain; 4 Institute of Biomedicine of Seville (IBIS), Virgen del Rocío Hospital, Seville, Spain; 5 Medical Oncology, Virgen de Valme Hospital, Seville, Spain; 6 UGC Emergency Service, Virgen del Rocío Hospital, Seville, Spain; 7 Diagnostic Imaging Unit, Radiology Service, Virgen del Rocío Hospital, Seville, Spain; 8 Methodology and Research Evaluation Unit, Hospital Virgen del Rocío, Seville, Spain; 9 Pharmacy, Hospital Virgen del Rocío, Seville, Spain

## Abstract

**Background:**

Optimal duration of anticoagulation for cancer-associated thrombosis (CAT) remains unclear. This study assessed D-dimer (DD) and high-sensitivity C-reactive protein (hs-CRP) levels after the withdrawal of anticoagulation treatment to predict the risk of venous thromboembolism (VTE) recurrence among patients with CAT.

**Methods:**

Prospective, multicentre study to evaluate CAT with ≥3 months of anticoagulation that was subsequently discontinued. Blood samples were taken when patients stopped the anticoagulation and 21 days later to determine the DD and hs-CRP levels. All patients were followed up for 6 months to detect VTE recurrence.

**Results:**

Between 2013 and 2015, 325 patients were evaluated and 114 patients were ultimately enrolled in the study. The mean age was 62 ± 14 years and nearly 40% had metastasis. Ten patients developed VTE recurrence within 6 months (8.8%, 95% confidence interval [CI]: 4.3–15.5%). The DD and hs-CRP levels after 21 days were associated with VTE recurrence. The subdistribution hazard ratios were 9.82 for hs-CRP (95% CI: 19–52) and 5.81 for DD (95% CI: 1.1–31.7).

**Conclusions:**

This study identified that hs-CRP and DD were potential biomarkers of VTE recurrence after discontinuation of anticoagulation in CAT. A risk-adapted strategy could identify low-risk patients who may benefit from discontinuation of anticoagulation.

## Introduction

The relationship between venous thromboembolism (VTE) and cancer is well known, with cancer being a major independent risk factor for VTE, being the second leading cause of death among patients with cancer.^1^ Furthermore, the incidence of VTE is steadily increasing among cancer patients, who have an elevated risk of VTE recurrence and bleeding.^2–5^ Anticoagulation treatment for 3–6 months has been shown to be safe and effective for patients with cancer and VTE,^6–10^ and the guidelines recommend first-line treatment for 3–6 months using low-molecular-weight heparin (LMWH) among patients with cancer-associated VTE.^11–13^ However, there are limited data regarding prolonged use of LMWH. Hokusai VTE Cancer is an open-label, non-inferiority trial that compared the use of edoxaban vs. dalteparin for the treatment of cancer-associated thrombosis (CAT) for at least 6 months and up to 12 months.^14^ The single-arm TiCAT and DALTECAN studies are the only prospective studies to evaluate the safety profiles of tinzaparin or dalteparin treatment for >6 months among patients with CAT.^15,16^ Nevertheless, it is possible that some patients with CAT might be able to tolerate withdrawal of anticoagulation treatment. However, there are no tools for the identification of patients with a low risk of VTE recurrence. The Cancer Duration of Anticoagulation based on Compression Ultrasonography Study (Cancer-DASCUS) evaluated residual deep vein thrombosis (DVT) to decide whether to withdraw or continue anticoagulation treatment, although that study’s findings did not change the guideline recommendations for the treatment of CAT.^17^

In cases with unprovoked VTE, the risk of VTE recurrence is greatest during the first year after withdrawal of anticoagulation treatment, and D-dimer (DD) tests and scores have been proposed to personalise anticoagulation treatment duration.^13,18^ In this context, DD is a degradation product of cross-linked fibrin, and high levels of DD indicate global coagulation activation and fibrinolysis among patients with malignancy and no thrombosis.^19,20^ Prospective studies have also demonstrated that DD levels can predict VTE recurrence among patients without cancer.^21–23^ Furthermore, levels of high-sensitivity C-reactive protein (hs-CRP) are significantly elevated among patients at risk for VTE, even in cases involving malignancy or inflammation.^24^ Nevertheless, existing prospective studies have not examined the clinical relevance of DD and hs-CRP levels for predicting VTE recurrence among patients with CAT. Therefore, the present multicentre prospective study evaluated DD and hs-CRP levels to determine whether they were associated with the risk of VTE recurrence. This information was then analysed in an attempt to identify patients who could tolerate the withdrawal of anticoagulation treatment based on a low risk of VTE recurrence.

## Patients and methods

### Design

This prospective multicentre study included patients with imaging-confirmed CAT (symptomatic or incidental) who underwent treatment with LMHW for ≥3 months, based on the guideline recommendations.^10,12,13^ A central, independent, adjudication committee (L.J.-P., A.S.-L. and R.O.-C.) reviewed all information related to cases with suspected VTE recurrence. This committee adjudicated VTE recurrences and evaluated fidelity and protocol compliance. The study protocol was approved by the Research Ethics Committee of Virgen del Rocio Hospital (NCT03134820) and complied with the regulations expressed in Spanish law 14/2007 regarding Biomedical Research. All patients provided written informed consent before enrolment. Patients were treated by specialists from the emergency, pulmonology, oncology, vascular surgery, and internal medicine divisions of three hospitals in Seville (Hospital Virgen Macarena, Hospital de Valme, and Hospital Virgen del Rocío).

To be considered eligible, patients had to fulfil the following criteria: (1) objectively diagnosed pulmonary embolism (PE) and/or DVT among patients with any active neoplasm (excluding non-melanoma skin cancer), (2) treatment using LMWH for ≥3 months after the diagnosis, (3) absence of residual vein thrombosis in DVT (defined as <0.3 cm), (4) absence of lupus anticoagulant (testing repeated twice at a 12-week interval), (5) absence of suspected chronic thromboembolic pulmonary hypertension (CTEPH), (6) absence of circumstances favouring treatment maintenance based on the clinician’s discretion, and (7) the patient provided written informed consent. The exclusion criteria were: (1) a life expectancy of <6 months, (2) cerebral metastases, (3) pregnancy, and (4) a lack of informed consent. Active neoplasm was defined as follows: (1) diagnosis of cancer in the 6 months prior to inclusion in the study; (2) having received any oncological treatment within the previous 6 months; and (3) presence of metastasis or cancer recurrence. Patients were actively interrogated to rule out CTEPH. We had suspicion of CTEPH if patients answered positively to any of the following questions: (1) Have you presented with dyspnoea while trying to conduct tasks that you could complete prior to the PE episode without problems? (2) Have you fully recovered from dyspnoea since your PE episode? (3) Have you presented with palpitations, chest tightness or fading without the known justification? (4) Have you had signs of congestive heart failure without the known aetiology or justification (jugular engorgement, peripheral oedema, or ascites)?

### Sample size

We assumed a recurrence rate of 10% during the first 6 months after discontinuation of anticoagulation treatment. Based on a safety level of 95% (1−*α*), statistical power of 90%, and a 15% mortality rate, the minimum required sample size was 113 patients.

### Anticoagulation plan and monitoring

Patients were treated from the first acute episode of VTE using LMWH at a weight-adjusted dosage according to the approved dose. The treatment continued as per the clinician’s discretion for ≥3 months. After 3 months of anticoagulation treatment, the clinician could decide to discontinue treatment based on the pre-determined criteria (Table 1). Blood samples were obtained from all patients to test for DD and hs-CRP on the day of anticoagulation withdrawal (baseline) and 21 days later. All blood samples were analysed in the same laboratory (H. Virgen del Rocio) using identical procedures. After the discontinuation of treatment, the patients were educated about and received written information regarding serious symptoms and were provided with a telephone number to contact the investigators if they had any concerns or developed suggestive symptoms. All patients were also followed up at 3 months and 6 months to evaluate their clinical status and complications (VTE recurrence, bleeding, or death). Episodes of VTE recurrence, bleeding, and all causes of death were recorded for analysis.

**Table 1:** Criteria for the withdrawal of anticoagulation treatment among patients with cancer-associated thrombosis

•At least 3 months of anticoagulation treatment
•Ultrasound-confirmed absence of residual venous thrombosis in the lower limbs (defined as <0.3 cm)
•Absence of cancer progression or signs that suggests non-stability of the disease
•Absence of lupus anticoagulant (testing repeated twice at a 12-week interval)
•Absence of suspected chronic thromboembolic pulmonary hypertension
•Absence of any circumstance favouring treatment maintenance based on the clinician’s discretion
•Informed consent received from the patient

### Blood samples and biomarker testing

Blood samples were obtained using antecubital venipuncture with minimal compression and a 21 G needle. At each sampling time (baseline and 21 days after treatment withdrawal), the first 3 mL of blood were discarded and then 21 plastic tubes with 0.109 M trisodium citrate (Vacutainer®, BD Biosciences, Erembodegem, Belgium) were filled. The samples were immediately sent to the laboratory, which performed testing for hs-CRP (N High Sensitivity CRP, Dade Behring®; normal range: <5 mg/L) on a Behring Nephelometer II System and for DD (Acute Care™ D-dimer test pack, Siemens Healthcare Diagnostics, Newark, DE, USA; normal range: <500 µg/L).

### Study variables

At enrolment, data were collected regarding the patients’ demographic characteristics, VTE risk factors, the VTE diagnosis (DVT and/or PE), anticoagulation treatment before inclusion, and oncological treatment before inclusion. The primary outcome was the ability of DD and hs-CRP (at baseline or after 21 days) to predict VTE recurrences 6 months after the discontinuation of anticoagulant treatment. The secondary outcomes were time from anticoagulation treatment withdrawal to VTE recurrence, all-cause mortality, and VTE-related mortality, which were determined using objective methods or via clinical consensus among the oncologists and pulmonologist.

Recurrent VTE was defined as suspected symptomatic (new or recurrent) PE with at least one of the following findings: (1) a new intraluminal filling defect in the sub-segmental or more proximal branches that was detected during spiral computed tomography; (2) a new intraluminal filling defect, the extension of an existing defect, or a new sudden occlusion of vessels with a diameter of >2.5 mm that was detected during pulmonary angiography; (3) a new perfusion defect (≥75% of a segment) with a local normal ventilation result (high probability) on the ventilation/perfusion lung scan; or (4) inconclusive findings from computed tomography, pulmonary angiography, or ventilation/perfusion lung scan but with compression ultrasonography or venography revealing new or extended DVT in the lower extremities. Suspected symptomatic (new or recurrent) DVT was defined as abnormal findings from compression or colour Doppler ultrasonography or an intraluminal filling defect detected during venography. Suspected symptomatic recurrent DVT was defined as (1) abnormal findings from compression ultrasonography with previous normal compression; (2) a substantial increase in diameter (≥4 mm) of the thrombus during full compression for previously non-compressible areas; or (3) extension of an intraluminal filling defect, a new intraluminal filling defect, or an extension of venous non-visualisation in the presence of a sudden cutoff during venography.

### Statistical analysis

Normally distributed continuous variables were reported as mean±standard deviation, and categorical variables were reported as number and frequency. The optimal levels ​​of DD and hs-CRP for predicting VTE recurrence were evaluated using a receiver operating characteristic (ROC) curve, and we evaluated whether the area under the curve was >0.5 based on the standard error obtained using DeLong’s method. In a second step, we evaluated the predictive values of these biomarkers using a multivariate logistic regression model with VTE recurrence as the dependent variable and metastasis as a covariate. Competing risk regression analysis of time to VTE recurrence (adjusted for metastatic disease) was performed for significant biomarkers (Fisher’s exact test, *p* < 0.05). Wald’s test was used to assess the effect of a variable within the competing risk regression model. All analyses were performed using the IBM SPSS software (version 20), EPIDAT software (version 4.1), and R software (version 3.0.1) with the “survival” and “cmprsk” packages.

## Results

**Fig. 1:**
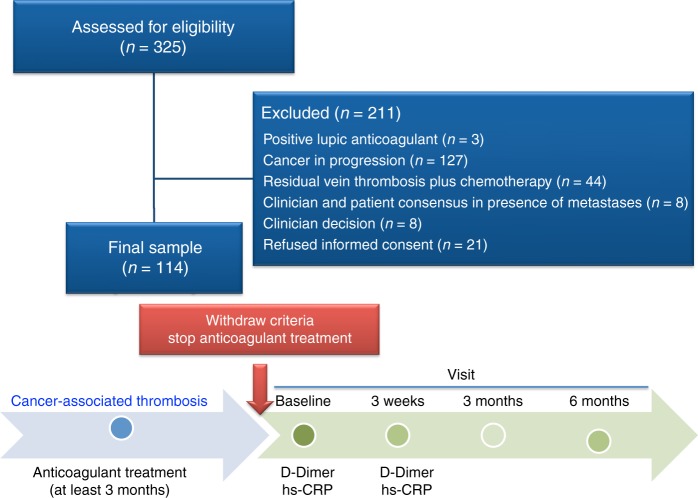
Flow diagram

Between December 2013 and October 2015, 325 patients were evaluated and 114 patients were ultimately enrolled in the study (Fig. 1). The characteristics of the excluded patients can be seen in supplementary table 1. The mean age of enrolled participants was 61.7 ± 13.7 years, and nearly 40% of the patients had metastases. VTE presented as follows: 53.5% (*n* = 61) DVT, 28.9% (*n* = 33) PE, 11.4% (*n* = 13) PE plus DVT, and 6.1% (*n* = 7) patients had atypical VTE (*n* = 4 in inferior vena cava and iliac territory, *n* = 2 upper limb DVT, and *n* = 1 splanchnic vein thrombosis). The patients’ baseline characteristics, clinical characteristics related to cancer, and cancer types are shown in Table 2. Six patients had bleeding prior to the discontinuation of anticoagulant treatment, two of whom had major bleeding. The median±range for the duration of anticoagulant treatment in all patients was 11.6 ± 54.7 months, with no differences between those with or without VTE recurrences (10.8 ± 33.38 vs. 11.7 ± 54.7 months; *p* = 0.88). We did not find an association between VTE recurrences and anticoagulation treatment for <12 months (6/61) vs. ≥12 months (4/53) (*p* = 0.75).

**Table 2:** Baseline characteristics of the included patients

	Total cohort (*n* = 114), *n* (%)	No VTE recurrence (*n* = 104), *n* (%)	VTE recurrence (*n* = 10), *n* (%)
Age, mean ± SD (years)	61.7 ± 13.7	61.8 ± 13.7	59.9 ± 14.3
Male, *n* (%)	58 (51.3)	51 (50)	7 (70)
Haematological cancer, *n* (%)	16 (14.3)	14 (13.9)	2 (20)
Solid tumour, *n* (%)			
Breast	20 (17.9)	18 (17.8)	2 (20)
Colorectal	20 (17.9)	20 (19.8)	0 (0)
Lung	12 (10.7)	9 (8.9)	3 (30)
Prostate	9 (8)	9 (7.9)	1 (10)
Bladder	6 (5.4)	6 (5.9)	0 (0)
Kidney	3 (2.7)	2 (2)	1 (10)
Pancreas	2 (1.8)	1 (1)	1 (10)
Brain	2 (1.8)	2 (2)	0 (0)
Others	22 (19.6)	21	0
Metastasis	45 (39.8)	39 (37.9)	6 (60)
ECOG performance status, *n* (%)			
0	56 (49.1)	52 (50.5)	4 (40)
1	51 (44.7)	46 (44.5)	5 (50)
2	3 (2.6)	3 (2.9)	0 (0)
3	1 (0.9)	1 (1)	0 (0)
4	3 (2.6)	2 (1.9)	1 (10)
VTE presentation, *n* (%)			
DVT	61 (53.5)	55 (53.4)	6 (60)
PE	33 (28.9)	33 (32)	0 (0)
DVT+PE	13 (11.4)	9 (8.6)	4 (40)
Atypical VTE location	7 (6.1)	7 (6.8)	0 (0)
Incidental VTE, *n* (%)	33 (29)	33 (32)	0 (0)
VTE recurrence	10 (8.8)	—	—
Death	10 (8.8)	6 (5.9)	2 (20)

*VTE* venous thromboembolism, *SD* standard deviation, *ECOG* Eastern Cooperative Oncology Group, *DVT* deep vein thrombosis, *PE* pulmonary embolism

During the 6 months after the withdrawal of anticoagulation treatment, 10 patients developed VTE recurrence (8.8%; 95% CI: 4.3–15.5%); however, none of these recurrences occurred in the first 21 days. Recurrence of VTE was significantly associated with the 21-day levels of DD (486 ng/mL vs. 1701 ng/mL; *p* = 0.002) and hs-CRP (2.5 mg/L vs. 8.4 mg/L; *p* = 0.002) (Table 3). Data regarding DD levels were available for 111 patients (97%), with a median 21-day value of 524 ng/mL (interquartile range: 374–1,126 ng/mL). Data regarding hs-CRP levels were available for 99 patients at baseline and 103 patients (90%) at 21 days, with a median 21-day value of 2.6 mg/L (interquartile range: 1.5–7 mg/L). After 21 days, normal values were observed for DD in 56% of patients (62/111) and for hs-CRP in 65% of patients (67/103). ROC curve analysis determined that the cutoff values for predicting 6-month VTE recurrence were 600 ng/mL for DD and 4.5 mg/L for hs-CRP. ROC curves for DD >600 ng/mL and for hs-CRP >4.5 mg/L 21 days after the withdrawal of anticoagulant treatment are provided in supplementary Fig. 1. VTE recurrences 6 months after the withdrawal of anticoagulant treatment in patients with DD ≤600 ng/mL (*n* = 62) vs. >600 ng/mL (*n* = 48) at day 21 was 1.6% (95% CI: 0.04–8.7%) vs. 18.8% (95% CI: 9–32.6%), respectively (*p* < 0.0001). VTE recurrences 6 months after the withdrawal of anticoagulant treatment in patients with hs-CRP ≤4.5 mg/L vs. >4.5 mg/L at day 21 was 1.5% (95% CI: 0.03–8.2%) vs. 33.3% (95% CI: 16.5–54%), respectively (*p* < 0.0001). The sensitivity, specificity, positive predictive value, and positive likelihood ratio values are shown in Table 4. We also calculated the biomarkers’ accuracy for predicting VTE recurrence 3 months after the discontinuation of anticoagulant treatment and found that the 21-day values were able to predict all recurrences (*n* = 9; 100%, 95% CI: 66–100%).

**Table 3:** Biomarkers according to VTE recurrence at 6 months

	Total cohort (*n* = 114), *n* (%)	No VTE recurrence (*n* = 104), *n* (%)	VTE recurrence (*n* = 10), *n* (%)	*p*
Metastasis	45 (39.8)	39 (37.9)	6 (60)	0.17
Biomarkers				
Baseline D-dimer (*n* = 111), median ng/mL (IQR)	333 (217–696)	327 (215–644)	615 (246–1112)	0.22
D-dimer at 21 days (*n* = 111), median ng/mL (IQR)	524 (374–1126)	486 (352–1046)	1701 (827–4034)	**0.002**
Baseline hs-CRP (*n* = 99), median mg/L (IQR)	2.7 (1.4–7)	2.4 (1.3–7.2)	4.6 (2.3–7.1)	0.28
hs-CRP at 21 days (*n* = 103), median mg/L (IQR)	2.6 (1.5–7)	2.5 (1.4–6.4)	8.4 (5.6–16.2)	**0.002**

*VTE* venous thromboembolism, *IQR* interquartile range, *hs-CRP* high-sensitivity C-reactive protein. Bold indicates that p was statistically significant. If you consider can be eliminated

**Table 4:** Predicting VTE recurrence using D-dimer and C-reactive protein at 3 weeks after withdrawing anticoagulation treatment

	Sensitivity, % (95% CI)	Specificity, % (95% CI)	Positive PV, % (95% CI)	Negative PV, % (95% CI)	Positive LHR, % (95% CI)	Negative LHR, % (95% CI)
VTE recurrence 3 months after the discontinuation of anticoagulant treatment						
Age-adjusted D-dimer (ng/mL)	100 (61–100)	61 (51.4–69.7)	12.8 (6–25.2)	100 (94.3–100)	2.56 (2.02–3.25)	0
D-dimer of >600 ng/mL	100 (61–100)	60 (50.4–68.7)	12.5 (6.59–24.7)	100 (94.3–100)	2.5 (1.98–3.16)	0
hs-CRP of >4.5 mg/L	100 (61–100)	69.1 (59.3–77.4)	16.7 (7.9–31.9)	100 (94.6–100)	3.23 (2.4–4.35)	0
D-dimer of >600 ng/mL and/or hs-CRP of >4.5 mg/L	100 (61–100)	50 (40.6–59.4)	10.2 (4.7–20.5)	100 (93.2–100)	2 (1.65–2.42)	0
Age-adjusted D-dimer and/or hs-CRP of >4.5 mg/L	100 (61–100)	59.1 (42.5–61.2)	10.5 (4.9–21.1)	100 (93.5–100)	2.08 (1.71–2.53)	0
VTE recurrence 6 months after the discontinuation of anticoagulant treatment						
Age-adjusted D-dimer (ng/mL)	90 (59.6–98.2)	62 (52.2–70.9)	19.1 (10.4–32.5)	98.4 (91.5–99.7)	2.37 (1.71–3.28)	0.16 (0.02–1.05)
D-dimer of >600 ng/mL	90 (59.6–98.2)	61 (51.2–70)	18.8 (10.2–31.9)	98.4 (91.4–99.7)	2.31 (1.67–3.18)	0.16 (0.03–1.07)
hs-CRP of >4.5 mg/L	90 (59.6–98.2)	71.7 (61.8–79.9)	25.7 (14.2–42.1)	98.5 (92–99.7)	3.18 (2.17–4.68)	0.14 (0.02–0.91)
D-dimer of >600 ng/mL and/or hs-CRP of >4.5 mg/L	90 (59.6–98.2)	51.5 (41.9–61)	15.5 (8.4–26.9)	98.1 (90.1–99.7)	1.86 (1.39–2.47)	0.19 (0.03–1.27)
Age-adjusted D-dimer and/or hs-CRP of >4.5 mg/L	90 (59.6–98.2)	53.5 (43.8–62.9)	16.1 (8.7–27.8)	98.2 (90.4–99.7)	1.93 (1.44–2.59)	0.19 (0.03–1.22)

*CI* confidence interval, *PV* predictive value, *LHR* likelihood ratio

The VTE recurrences involved DVT alone (*n* = 8), PE (*n* = 1), and superficial vein thrombosis (*n* = 1) with involvement of the safeness vein very close to the common femoral vein. The clinical characteristics of these patients are shown in supplementary Table 2. The cancer types for these patients were lung (*n* = 3), haematological (*n* = 2), breast (*n* = 2), kidney (*n* = 1), prostate (*n* = 1), and pancreas (*n* = 1). Supplementary figure 2 shows a double axis plot of the VTE recurrences with the corresponding 21-day values for DD and hs-CRP. After discontinuation of anticoagulant treatment, two patients had bleeding, one of whom had major bleeding.

**Fig. 2:**
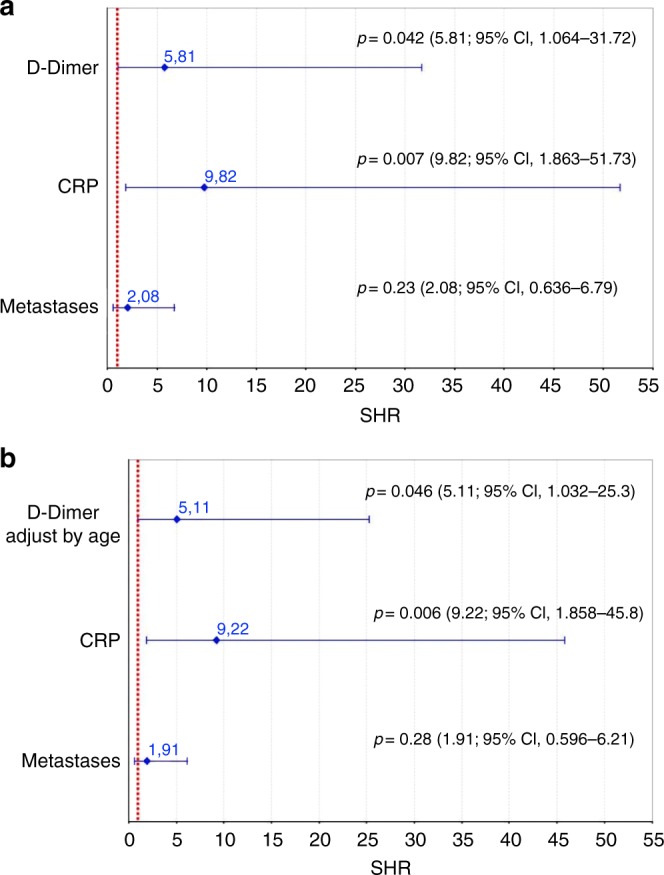
Subdistributional hazard ratios (SHRs and 95% CIs) in the combined competing risk regression model. Competing risk regression analysis of time to VTE recurrence was performed after accounting for the study design variables and significant predictors from the individual analyses. **a** The SHRs for metastasis, hs-CRP of >4.5 mg/L, and D-dimer of >600 ng/mL. **b** SHR for metastasis, hs-CRP of >4.5 mg/L, and age-adjusted D-dimer (500 ng/mL for ≤50 years or age × 10 ng/mL for >50 years). SHR subdistributional hazard ratios, CI confidence interval, hs-CRP high-sensitivity C-reactive protein

Competing risk analysis of the time to VTE recurrence, which was adjusted for metastatic disease, revealed that the 21-day values for hs-CRP and DD remained significantly associated with the risk of VTE recurrence. The subdistribution hazard ratios (SHRs) were 9.82 for hs-CRP >4.5 mg/L (95% CI: 1.86–51.7; *p* = 0.007) and 5.81 for DD >600 ng/mL (95% CI: 1.06–31.72; *p* = 0.042). We also calculated the SHR for age-adjusted DD levels (age × 10 ng/mL), which revealed a similar result (Fig. 2).

## Discussion

The present study revealed that elevated levels of DD (>600 ng/mL) and hs-CRP (>4.5 mg/L) at 21 days after stopping anticoagulation treatment could predict VTE recurrence among patients with CAT. These cutoff values provided 100% (95% CI: 66–100%) sensitivity and 90% (95% CI: 60–98%) sensitivity for VTE recurrence 3 and 6 months after the withdrawal of anticoagulant treatment. Based on these results, approximately 66% of patients could safely stop anticoagulation treatment based on their 21-day hs-CRP values and 56% of patients could safely stop treatment based on their DD values. In VTE, it is generally recommended to stop anticoagulant treatment when the risk of recurrence in the first year is below 5–8%.^25,26^ The annual risk of VTE recurrences in cancer patients is 15%.^13^ In this study, 6-month VTE recurrence was 8.8% (95% CI: 4.3–15.5%), which decreased to 1.6% (95% CI: 0.04–8.7%) or 1.5% (95% CI: 0.03–8.2%) if DD was ≤ 600 ng/mL or hs-CRP was ≤ 4.5 mg/L at 21 days, respectively. These results highlight the importance of determining the appropriate duration of treatment for CAT.

There are limited data regarding the appropriate duration of anticoagulation treatment for CAT. The DACUS study evaluated patients with proximal DVT or PE and active cancer who received 6 months of LMWH treatment and subsequently had residual DVT at the 6-month ultrasonography examination.^17^ These patients were randomised to either stop treatment or receive 6 additional months of LMWH therapy, with follow-up for 12 months. The 6 additional months of LMWH reduced the rate of VTE recurrence, although the difference was not statistically significant between the group that stopped treatment (22%, 95% CI: 15–30%) and the group that continued treatment (15%, 95% CI: 9.2–22.9%; *p* = 0.18). The same study revealed that stopping LMWH treatment after 6 months was associated with a low risk of VTE recurrence within 12 months among patients without residual DVT (2.8%, 95% CI: 0.6–8.1%).^17^ However, despite these findings, the guideline recommendations for managing the risk of VTE recurrence have not changed. Moreover, residual venous thrombosis in unprovoked VTE has been widely evaluated; however, it is only considered a mild risk factor for VTE recurrence and this variable is not included in any scores for predicting recurrence among patients with unprovoked VTE.^27^ Therefore, additional data are needed to better understand the role of residual venous thrombosis in cancer-associated VTE.

A previous study examined the relationship between CRP and DD,^28^ which revealed that the levels of CRP and DD were correlated (*r* = 0.64, *p* < 0.01) and significantly elevated among patients with DVT (*p* < 0.001). Furthermore, among patients with suspected DVT, plasma CRP levels were significantly associated with the presence of DVT (*p* < 0.001), malignancy (*p* < 0.001), and inflammatory disease (*p* = 0.009).^28^ Moreover, previous studies have evaluated the value of CRP and DD levels for predicting VTE among patients with cancer.^24,29^ Kroger et al. evaluated prospectively collected data from 507 patients with cancer, which revealed a 12% rate of VTE during follow-up, which was significantly associated with inpatient treatment, prior VTE, familiar history of VTE, chemotherapy, fever, and CRP levels.^24^ Ay et al. in a prospective observational study revealed that the rate of VTE occurrence was 7.6% among 821 patients with cancer; this was independently predicted by DD and prothrombin fragment 1+2 levels (in the 75th percentile).^29^ These studies highlight the value of CRP and DD levels in predicting VTE among patients with CAT, suggesting that it is plausible to use these biomarkers to predict VTE recurrence during follow-up. Khorana et al. evaluated the role of biomarkers for the prediction of recurrent VTE during anticoagulant treatment in patients with CAT.^30^ In a post hoc sub-analysis from the CATCH trial, circulating tissue factor (TF), DD, soluble P-selectin, Factor VIII, and CRP were analysed in 900 patients. The only potential biomarker identified was TF in the uppermost quartile (SHR: 3.3; 95% CI: 1.7–6.4); venous compression (SHR: 3.1; 95% CI: 1.4–6.5) and hepatobiliary cancer diagnoses (SHR: 5.5; 95% CI: 2.3–13.6) were identified as clinical risk factors. Further validation of this approach is necessary.

Cancer patients differ substantially in terms of type, stage, and histology, which suggests that treatment duration should vary as well.^31^ Furthermore, a personalised approach to anticoagulation treatment, based on biomarkers or scores, has been investigated in non-cancer populations.^13,18^ Thus the present study adds value because, although clinicians know when to start anticoagulation treatment for patients with cancer, there is no consensus regarding the appropriate treatment duration. Nevertheless, as in unprovoked VTE, it is crucial to identify patients who can tolerate treatment withdrawal. Our work revealed that the 6-month incidence of VTE recurrence was high (8.8%; 95% CI: 4.3–15.5%), which suggests that additional research is needed to investigate biomarkers that can be used to identify patients with low or high risks of VTE recurrence and guide their treatment. It would be interesting to adjust for the type of cancer in future studies; unfortunately, the sample size in this study did not allow us to do so.

The present study has several limitations. First, a potential pre-selection study bias could be considered. However, we were attempting to select a subgroup of patients who could benefit from the discontinuation of anticoagulant treatment. Second, one of the criteria to determine whether anticoagulant treatment could be stopped was the absence of any circumstance favouring treatment maintenance based on the clinician’s discretion. These criteria are subjective; clinicians consider a variety of other reasons as to why anticoagulation should be continued. Although these criteria could be considered a weakness, there are other scores in which clinician subjectivity are considered, for example the Hestia criteria or the Wells score, which have a good reproducibility.^32,33^ Third, broad confidence intervals were calculated for sensitivity and the SHR, which is related to the small number of recurrences. The small sample size is likely related to the limited number of participating centres. Fourth, the study sample had heterogeneous characteristics in terms of cancer location, oncological treatment, and VTE location. The influences of these variables can only be evaluated in a larger sample of patients or studies with more strict inclusion criteria. No one with incidental VTE had VTE recurrences, although there was no relationship between incidental VTE and VTE recurrences (*p* = 0.06). This finding should be investigated in future studies.

DD and hs-CRP at 21 days after the discontinuation of anticoagulation were associated with the risk of VTE recurrence in patients with CAT. Biomarkers, such as DD and hs-CRP, may play a role in determining the optimal anticoagulant treatment duration in patients with CAT.

## Electronic supplementary material

Supplementary Table 1

Supplementary Table 2

Supplementary Figure 1

Supplementary Figure 2

Supplementary Figures legend

## Appendix

HISPALIS group: Seville, Spain: L. Jara-Palomares, A. Solier-Lopez, T. Elias-Hernandez, M. I. Asensio-Cruz, I. Blasco-Esquivias, V. Sanchez-Lopez, E. Arellano-Orden, L. Suarez-Valdivia, M. Rodriguez de la Borbolla, A. Ruiz-GarcIa, E. Montero-Romero, S. Navarro-Herrero, J. L. Lopez-Campos, M. P. Serrano-Gotarredona, J. M. Praena-Fernandez, J. M. Sanchez-Diaz, S. Marin-Romero, R. Otero-Candelera.

## **Availability of data and materials:**

Data available upon request.
