# Supplementary material for: D-dimer and high-sensitivity C-reactive protein levels to predict venous thromboembolism recurrence after discontinuation of anticoagulation for cancer-associated thrombosis
Source: Br J Cancer. 2018 Oct 15;119(8):915–21. doi: 10.1038/s41416-018-0269-5 (PMC6203717; doi:10.1038/s41416-018-0269-5)
Supplement: Supplementary file 1 — Supplementary Table 1 [file 41416_2018_269_MOESM1_ESM.doc]

**Supplementary Table 1. Baseline characteristics of the patients excluded from the study**

| **Characteristic** | **Total cohort (n=211)**  **n (%)** |
| --- | --- |
| Age, mean ± SD (years) | 62.8 ± 13.3 |
| Male, n (%) | 109 (55.3) |
| Weight, mean ±SD (kg) | 76.2 ± 15.24 |
| Hematologic cancer n (%) | 16 (7.6) |
| **Solid tumour, n (%)** |  |
| Lung | 31 (14.7) |
| Breast | 25 (11.8) |
| Colorectal | 39 (18.5) |
| Kidney | 6 (2.8) |
| Bladder | 12 (5.7) |
| Ovarian | 13 (6.2) |
| Prostate | 6 (2.8) |
| Pancreas | 7 (3.3) |
| Brain | 3 (1.4) |
| Others | 47 (22.3) |
| **ECOG performance status score, n (%)** |  |
| 0 | 47 (22.3) |
| 1 | 106 (50.2) |
| 2 | 25 (11.8) |
| 3 | 4 (1.9) |
| 4 | 11 (5.2) |
| **VTE presentation, n (%)** |  |
| DVT | 77 (36.5) |
| PE | 55 (26.1) |
| DVT + PE | 43 (20.4) |
| Unusual location | 21 (10) |
| Incidental VTE, n (%) | 59 (28%) |
| VTE: venous thromboembolism; SD: standard deviation; DVT, deep vein thrombosis; PE, pulmonary embolism. | |
