# Supplementary material for: D-dimer and high-sensitivity C-reactive protein levels to predict venous thromboembolism recurrence after discontinuation of anticoagulation for cancer-associated thrombosis
Source: Br J Cancer. 2018 Oct 15;119(8):915–21. doi: 10.1038/s41416-018-0269-5 (PMC6203717; doi:10.1038/s41416-018-0269-5)
Supplement: Supplementary file 2 — Supplementary Table 2 [file 41416_2018_269_MOESM2_ESM.docx]

**Clinical characteristics of patients with VTE recurrences.**

| **Patient** | **Time to event** | **Age** | **Sex** | **VTE type** | **Cancer location** | **Metastases** | **ECOG** | **hs-CRP 21 days** | **D-Dimer 21 days** | **Type VTE recurrence** |
| --- | --- | --- | --- | --- | --- | --- | --- | --- | --- | --- |
| **1** | 21 | 66 | Male | DVT | Lung | Yes | 1 | 4.7 | 5,015 | DVT |
| **2** | 26 | 76 | Male | DVT | Hematologic | Yes | 1 | 48.3 | 18,668 | DVT |
| **3** | 34 | 80 | Male | DVT - PE | Kidney | Yes | 1 | 9.7 | 1,216 | DVT |
| **4** | 42 | 53 | Female | DVT | Breast | No | 0 | 6.1 | 3,708 | DVT |
| **5** | 42 | 61 | Male | DVT - PE | Lung | No | 0 | 7.0 | 2,177 | DVT |
| **6** | 91 | 41 | Male | DVT | Hematologic | Yes | 0 | 16.2 | 621 | SVT* |
| **7** | 102 | 67 | Female | DVT - PE | Breast | No | 0 | 5.9 | 896 | DVT |
| **8** | 117 | 34 | Female | DVT | Lung | Yes | 1 | 16.0 | 1,226 | DVT |
| **9** | 125 | 61 | Male | DVT | Prostate | No | 1 | 2.4 | 266 | DVT |
| **10** | 203 | 60 | Male | DVT - PE | Pancreas | Yes | 3 | 16.3 | 3,151 | PE |
| VTE: Venous thromboembolism; ECOG: Eastern Cooperative Oncology Group performance status; hs-CRP: hs-CRP: high sensitivity C-reactive protein; DVT: deep vein thrombosis; PE: pulmonary embolism. | | | | | | | | | | |
