# Supplementary figures and images for: D-dimer and high-sensitivity C-reactive protein levels to predict venous thromboembolism recurrence after discontinuation of anticoagulation for cancer-associated thrombosis
Source: Br J Cancer. 2018 Oct 15;119(8):915–21. doi: 10.1038/s41416-018-0269-5 (PMC6203717; doi:10.1038/s41416-018-0269-5)

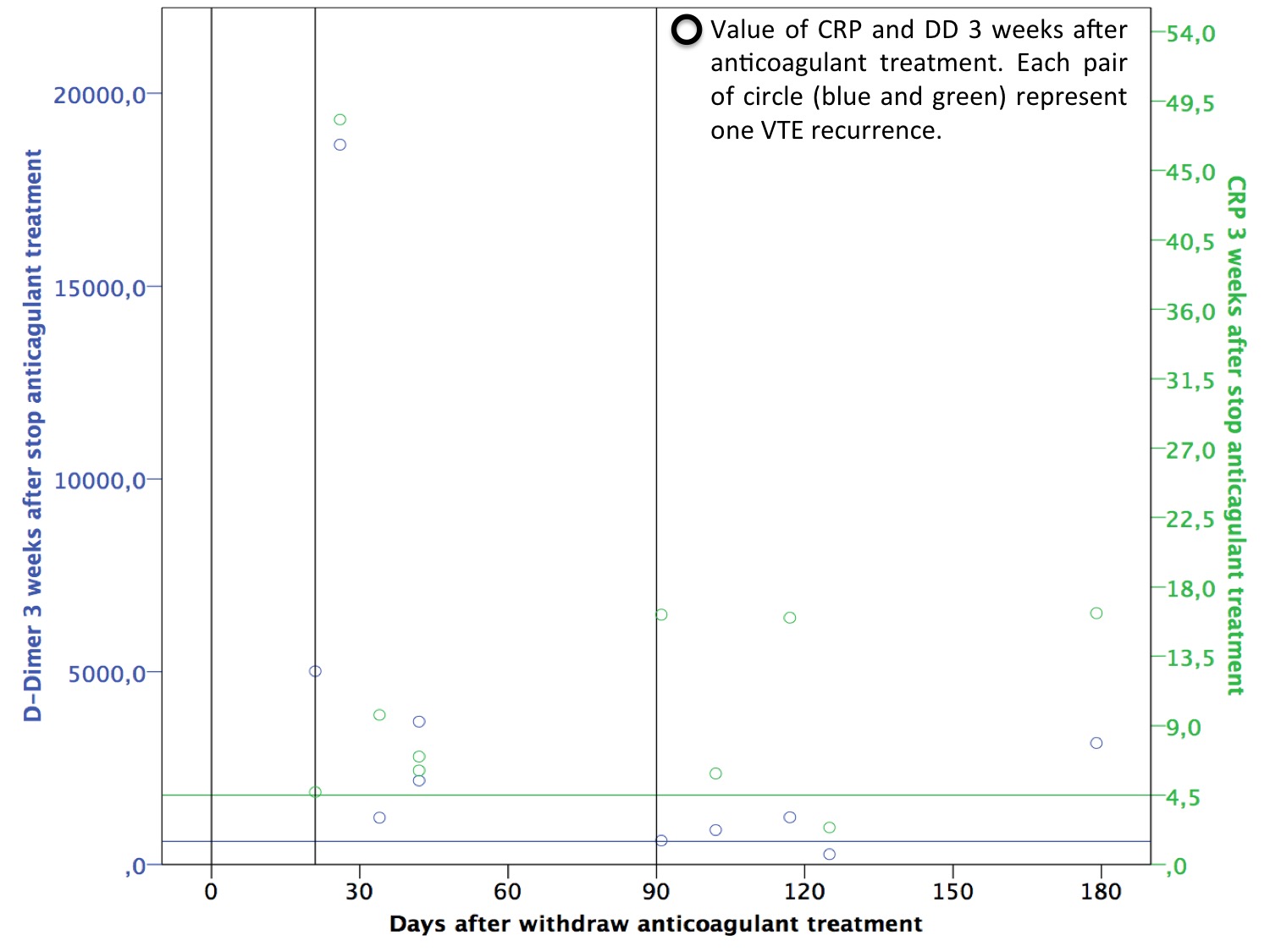

Supplement: Supplementary file 4 — Supplementary Figure 2 [file 41416_2018_269_MOESM4_ESM.jpg]
