# Supplementary material for: D-dimer and high-sensitivity C-reactive protein levels to predict venous thromboembolism recurrence after discontinuation of anticoagulation for cancer-associated thrombosis
Source: Br J Cancer. 2018 Oct 15;119(8):915–21. doi: 10.1038/s41416-018-0269-5 (PMC6203717; doi:10.1038/s41416-018-0269-5)
Supplement: Supplementary file 5 — Supplementary Figures legend [file 41416_2018_269_MOESM5_ESM.docx]

**Supplementary Figure 1. Receiver operating characteristic (ROC) curve for DD >600 ng/mL and for hs-CRP >4.5 mg/L 21 days after the withdrawal of anticoagulant treatment.**

**Supplementary Figure 2. Double axis plot showing the C-reactive protein and D-dimer values at 3 weeks after stopping anticoagulation treatment.**

Each pair of blue and green circles represent a single case of VTE recurrence.
